# Laparoscopic cholecystectomy in patients aged 60 years and over – our experience

**Published:** 2016

**Authors:** D Serban, C Branescu, C Savlovschi, AP Purcărea, A El-Khatib, SA Balasescu, A Nica, AM Dascalu, G Vancea, SM Oprescu, C Tudor

**Affiliations:** * Carol Davila” University of Medicine and Pharmacy, Bucharest, Romania; ** Upper Digestive Surgery Clinic, Emergency University Hospital, Bucharest, Romania

**Keywords:** laparoscopy, acute cholecystitis, elderly, cholecystectomy

## Abstract

**Aim.** To analyze the efficiency of laparoscopic cholecystectomy for the population aged 60 years and over admitted with acute cholecystitis, the clinical features and associated pathology presented by these patients and the impact of these factors on the choice of surgical technique.

**Materials and method.** A retrospective study was carried out between February 2010 and February 2015, on patients aged 60 years and over, operated in emergency for acute cholecystitis in our clinic. All data were extracted from the registered medical documents and operatory protocols.

**Results.** A total of 497 surgeries were performed for acute cholecystitis, of which 149 were patients aged 60 years and over (30%). Open surgery is much better represented in the population aged over 60 years (61.75% vs. 29.98%). One major cause is the associated pathology that increases the anesthetic risk and hampers a laparoscopic procedure. The conversion rate in the study group presented a higher percentage, but not more exaggerated than in the general population (6.71% vs. 4.63 %).Patients who underwent laparoscopic surgery had a faster recovery and required lower doses and shorter term pain medication, in contrast to conventional surgery (1,8 days vs. 5.7 days). Bile leak has been of reduced quantity, short-term and stopped spontaneously. Only one case needed reintervention, in which aberrant bile ducts that were clipped were found in the gallbladder bed, was operated by laparoscopy. Wound infections and swelling were also encountered more frequently in patients that underwent classic surgery (3.24%).

**Conclusions.** Performing laparoscopic cholecystectomy, when possible, has produced very good results, reducing the average length of stay of patients and even decreasing the number of postoperative complications, thus allowing a faster reintegration of patients into society. The main concern was related to the associated pathology that increased the anesthetic risk.

## Introduction

Cholelithiasis is the most frequent biliary pathology, with potentially high severity, especially by the presence of its complications. One of these is the acute cholecystitis, a frequent reason for an emergency presentation to the hospital. What should be mentioned in the case of patients older than 60 years is the higher frequency of associated pathologies that can influence the type and effectiveness of treatment.

The aim of this paper is to analyze the advantages and disadvantages of the laparoscopic technique for the population aged over 60, the clinical features and associated pathology presented by these patients and the impact of these factors on the choice of surgical technique. The study tried to answer to the question whether the technique of laparoscopic cholecystectomy is safe for the population aged over 60 years and what its advantages are.

## Materials and methods

A retrospective study was carried out between February 2010 and February 2015, on patients aged over 60 operated in emergency in our clinic for acute cholecystitis. All the data were extracted from registered medical documents and operatory protocols. Anamnesis aimed at revealing the symptoms for admission, disease history and co-existing pathology. The clinical examination was meant to highlight the presence or the absence of fever, chills,characteristic pain located in the right upper abdominal quadrant, jaundice, and signs of peritoneal reaction. All the patients underwent routine paraclinical exams (the total count of blood cells and leukocyte profile, bilirubin, alanine aminotransferase (ALT), aspartate aminotransferase (AST), amylase, blood sugar, creatinine and urea). Ultrasonography was used to confirm cholelithiasis, local inflammatory changes and to evaluate the possibility of a coexisting choledocholithiasis.The frequency of performance of laparoscopic cholecystectomy versus open surgery, the conversion rate and the comparative results obtained by the two techniques, in terms of complications, mortality and average length of stay, were analyzed.

## Results

A total of 497 surgeries for acute cholecystitis were performed in the study period, of which 70.02% were operated laparoscopically and 29.98% classically, the conversion rate being of 5.46%. Out of the total number of 497 patients, 149 were aged over 60 years, i.e. 30% of the total. Of these, 115 were women (77.18 %) and 34 men (22.82%), the sex ratio being consistent with the existing data in the field, confirming the more frequent occurrence of gallstones in women.

**Fig. 1:**
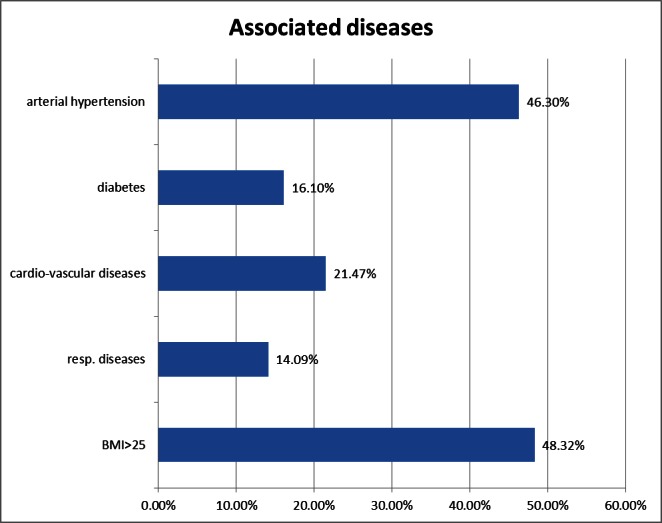
Co-existing pathology in the study group

The most frequently encountered symptoms at admission were the following: right upper quadrant pain in 71.14% of the cases, nausea and vomiting in 51.67% of the cases, fever, or chills in 48.32%, and jaundice in 16.1% of the cases. The co-existing pathology in the study group is presented in **Fig. 1**. 

The presence of a previous abdominal surgery in the patients’ history was a contraindication for a laparoscopic approach. This was noted in 31.30% of the women and 38.23% of the men included in the study group.

Preoperative preparation of thestudied patients did not differ depending on the surgical technique used, consisting in: fasting, hydroelectrolytic rebalancing, prophylactic antibiotics, antiemetic medication, and analgesics.

Out of the 149 patients over 60 years of age admitted for acute cholecystitis, 92 were operated classically (61.75%), 47 laparoscopically (31.54%) and, in 10 cases (6.71%), the conversion was imposed. 

**Fig. 2:**
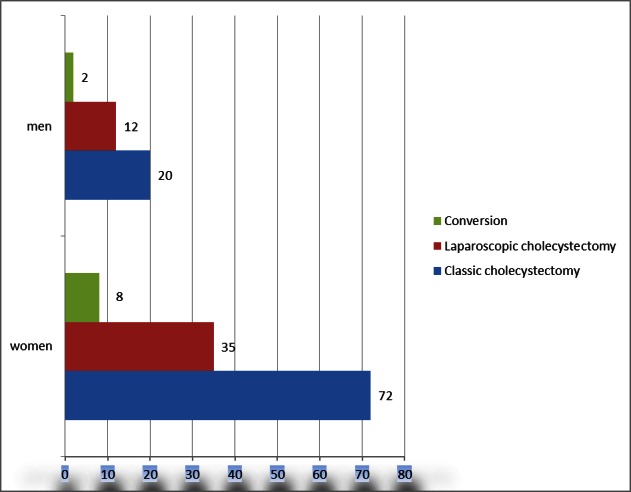
Classical vs. laparoscopic approach according to sex ratio in the study group

**Fig. 3:**
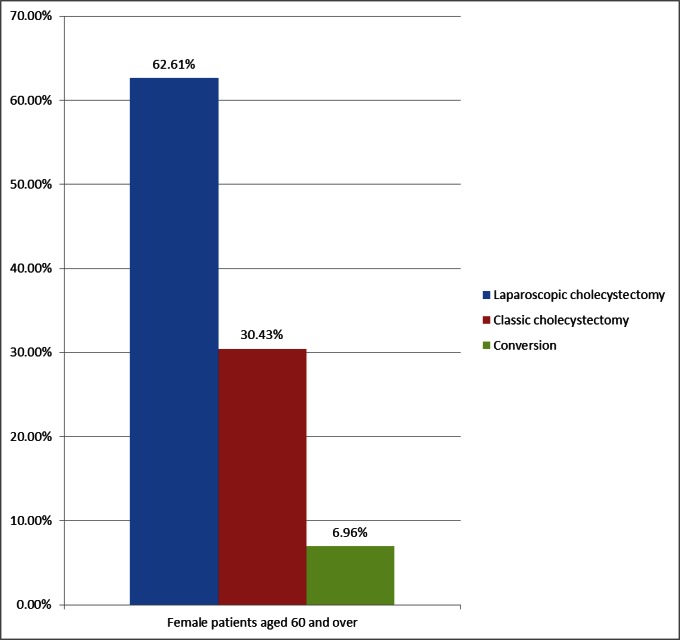
Classical vs. laparoscopic approach in women aged over 60 with acute cholecystitis

**Fig. 4:**
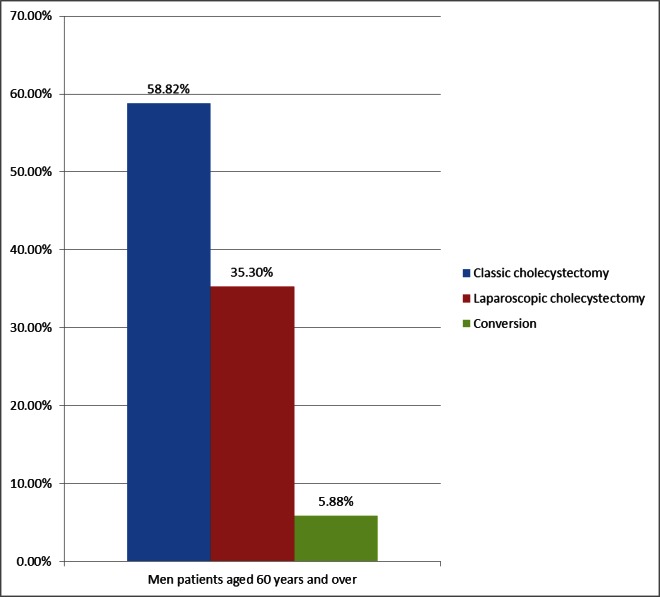
Classical vs. laparoscopic approach in men aged over 60 with acute cholecystitis

Classical surgical technique is much better represented in the population aged over 60 years: 61.75% vs. 29.98% in the general population. One major cause was the associated pathology that increasedthe anesthetic risk and hampered a laparoscopic procedure.

In the group of 92 cholecystectomies performed by a classical approach, the anatomopathological forms were catarrhal (40.22%), phlegmonous (29.35%), gangrenous (18.48%) and, in 11.95% of the cases a pericholecystic plastron was encountered. Of the 47 laparoscopic operations, 65.95% were catarrhal and 35.05% phlegmonous. What should be taken into account is that laparoscopy is not indicated in cases of suspected pericholecystic inflammatory mass due to the increased risk of damage to the surrounding structures.

The distribution of open surgery, laparoscopic approach and the conversion rate did not show significant variations between the group of women and men (**Fig. 4**). The conversion rate in the study group presented a higher percentage, but not more exaggerated than in the general population (6.71% vs. 4.63%). The reasons were the following:

-anatomical variations and obscure anatomy as a result of acute inflammation (60%),which did not allow a proper viewing of the Calot triangle and consequently made it impossible to complete the operation by a laparoscopic technique

- profuse bleeding (30%), which could not be controlled laparoscopically

- cardiovascular or respiratory decompensation during surgery (10%), which resulted into a conversion to open surgery

The main operative incidents encountered were haemorrhage and iatrogenic perforation of the gallbladder (**Table 1**). The second incident was more troublesome than serious, especially when the grasping and extraction of the lost gallstones in the peritoneal cavity was necessary, that maneuver prolonging the operation.

**Table 1:** Intraoperatory incidents and reinterventions: laparoscopic vs. classical approach

	Intraoperatory incidents	Reinterventions
Laparoscopic cholecystectomy	bleeding (8 cases) gallbladder effraction (11 cases)	bile leakage (1 case) : clip subhepatic abscess (1 case): laparoscopy - laparotomy
Open cholecystectomy	bleeding (12 cases) gallbladder effraction (5 cases)	evisceration (1 case) dynamic ileus (1 case)

The mean postoperative hospital stay was of 3.4 days in the laparoscopic group and of 7.9 days in the classical cholecystectomy group. Postoperative care consisted of maintaining a correct hydroelectrolytic balance, antibiotics, analgesics, anti-secretory and spasmolytic parenteral medication. In the cases of laparoscopic surgery, patients required lower doses and shorter-term pain medication, in contrast withthe conventional surgery (a mean period of 1,8 days vs. 5.7 days). Resumption of bowel movements was faster in the case of patients receiving laparoscopic cure, as opposed to the other group. Accordingly, the resumption of oral food intake was faster in these patients.

98.66% of the patients had a favorable outcome. There were two deaths, both operated by open surgery: one was due to pulmonary embolism and one due to heart failure. The two deaths were observed in female patients, and were due to the advanced age and associated comorbidities, with a high implicit associated operatory risk. 

The bile leak observed during the monitoring of the drainage tube was of reduced quantity, short-term and stopped spontaneously. Only one case operated by laparoscopy needed reintervention, in which aberrant bile ducts that were clippedwere found in the gallbladder bed. Monitoring the drainage tube was also necessary for the assessment of any bleeding episodes. No significant differences were found between the two studied groups. Wound infections and swelling were also encountered more frequently in patients who underwenta classical surgery (3.24%) (**Table 2**).

**Table 2:** Postoperative complications: laparoscopic vs. classical cholecystectomy

	Laparoscopic cholecystectomy (47 CASES)	Classical cholecystectomy (92 cases)
Limited bile leakage	4 (8.5%)	10 (10.86%)
Choleperitoneum	0	1 (1.08%)
Perihepatic fluid collections	1 (2.12%)	1 (1.08%)
ileus	0	1 (1.08%)
Parietal infections	0	3 (3.24%)
Eventration/ evisceration	0	1 (1.08%)

## Discussions

The use of laparoscopic cholecystectomy in older patients is complicated by comorbid conditions that are concomitant with the advanced age and may increase postoperative complications and the frequency of conversion to open surgery [**1**].Several clinical studies showed that laparoscopy is underused in elderly, due to the concerns of the physiologic demands of laparoscopic surgery, which derive from carbon dioxide pneumoperitoneum, transient increased intra-abdominal pressure, and patient positioning. The mechanical and metabolic demands of CO2 pneumoperitoneum include acid-base and blood gas disturbances, alterations to pulmonary and cardiovascular physiology and splanchnic and renal hypoperfusion. Pneumoperitoneum decreases functional residual capacity, lung compliance, and peak airway pressures, and absorbed intraperitoneal CO2 causes hypercarbia and acidemia. Both increased intra-abdominal pressure and reduced cardiac function, risk renal and hepatic hypoperfusion resulting in oliguria and transient hepatocellular injury [**2**-**5**].

Another important aspect concerning the intraoperative difficulty is the local situation. Patients older than 60 years presented to the hospital with symptoms of acute cholecystitis after a long period,during which they suffered repeated episodes of biliary colic. These episodes might have consequences on the anatomical structures, causing sclerosis and adhesions between the gallbladder and the surrounding connective tissue, due to repeated inflammation. Even patients who did not undergo previous abdominal surgery may present these anatomical fibrous reshufflings. Therefore, the local situation found intraoperatively, may hinder the advance of the operation, something that poses difficulty for both the surgeon and the patient, who is subject to additional stress and increased operation time. Chronic antiaggregant or anticoagulant medication in addition to local sclerosis may lead to uncontrolled bleeding and difficulties in a correct view of the surgical anatomical elements, imposing conversion to open surgery.

Even though elderly patients are more likely to present with several co-morbidities in advanced stages, early laparoscopic cholecystectomy for elderly patients with acute gallbladder disease proved to be safe and effective, and should be regarded as the standard of care, with the condition of an appropriate selection of the cases in this patient population [**6**-**8**].

Other studies proved that age > 65 years, male gender, acute cholecystitis, thickened gallbladder wall, diabetes mellitus, ASA 3 and previous upper abdominal surgery were significantly associated with an increased risk of conversion. Evaluating these factors was useful for the doctors to make a suitable operation scheme [**9**-**11**].In patients aged 80 or older, the rates of acute cholecystitis, conversion to open surgery, and postoperative complications were significantly higher than in other groups [**12**].

Laparoscopic surgery can be safely applied without further increasing the surgical risks and age alone should not be considered a contraindication to laparoscopic cholecystectomy. The complications can be minimized by carefully selecting the patients and by experienced teams with high technical capabilities operating on such patients [**12**-**14**].

On the other hand, old patients are the ones who have the most to gain from the laparoscopic techniques. The enhanced recovery offered by laparoscopy, with reduced postoperative pain, improved mobilization, shorter hospital length of stay and fewer complications may be most advantageous in this group with a prevalent comorbidity and reduced physiologic reserve [**15**,**16**]. The neuroendocrine and inflammatory responses lower than those of conventional cholecystectomy may also contribute to the lower morbidity of laparoscopic cholecystectomy [**17**].

## Conclusions

For patients over 60 years, who presented with symptoms of acute cholecystitis, the classical surgical technique was used more often than in the general population. The conversion rate was higher for the population chosen for study than for the general population, most likely due to associated diseases of patients in the study.

Performing laparoscopic cholecystectomy, when possible, has produced very good results, reducing the average length of stay of patients and even decreasing the number of postoperative complications, thus allowing a faster reintegration of patients into the society.

Although laparoscopic cholecystectomy is a new gold standard in resolving surgical pathology lithiasic, resorting to this technique in patients over 60 years, is a decision that should be carefully weighed, taking into account comorbidities of patients, clinical atypical features, anatomoclinicalforms and the possible necessity of conversion.
